# Report of Cases in Dr. Hamilton’s Surgical Dispensary at the Medical Department of the Buffalo University

**Published:** 1848-06

**Authors:** 


ART. III.—*Report of Cases in Dr. Hamilton's Surgical Dispensary at the Medical Department of the Buffalo University.*

*Saturday March 5th.—Tertiary Syphilis.*—Woman, aged about 40. Has had chancres and buboes; does not know whether she was treated with mercury or not; had chancres nearly three years since. Has had ulcers on various parts of the body since, and sore throat; and has now a constant and intense pain in the head, apparently in bones of cranium. ℞.—Iodide of Potash, ʒviii; Decoct. of Sarsaparilla ℥xv, of which she is to take a teaspoonful three times a day.

*Tertiary Syphilis.*—Man, aged about 45. Three years since had chancres and buboes, treated with mercury, was apparently cured. Two years after severe pains commenced in the bottom of one foot and in the top of the head; had extensive ulcers in pharynx and nose, with copious discharges from the latter. Under the care of Dr. King, who gave him iodide of potash, he improved rapidly, and nothing now remains but a slight eruption on head and arms. He was advised to take, for a few days the deuto-iodide of mercury, carrying it to the production of a slight ptyalism, and then resume the iodide of potash.

*Foreign Syphilis*, or scrofula modified by a hereditary taint. Girl aged about 6 years. Has an enlarged knee, ulcers on various parts of the body, and copper colored spots. She was advised to use tincture of iodine, &c.

*Wednesday, March* 9—*Comminuted Fracture of Clavicle.*— —— aged 29 years, was thrown yesterday from the back end of a wagon and fell upon his shoulder; right clavical is broken at two points, the intercepted piece being about an inch and a half in length and displaced.

It was noticed first, that contrary to the general opinion of surgeons in such fractures, the patient could readily raise his hand to his head, and such in fact is usually the case: and next that by no force or position could the fragments be made to resume their original situation, and the patient was accordingly notified that a deformity was inevitable. Dr. Hamil' ton discussed the various modes of dressing fractured clavicles, and stated his preference for what is called Fox's apparatus. Yet he admitted that with this he did not generally succeed in fractures occurring in adults in preventing deformity. Nor were other kinds of apparatus more successful, a statement which he confirmed by the following remarks from Velpeau, "with all the bandages imaginable, we cannot prevent oblique fracture of the two internal thirds from leaving a deformity."

The patient was told, however, that he might perhaps find some consolation for his misfortune in the reflection that William III, King of England, died from a broken clavicle, just 147 years to a day (8th day of March 1701) before, this patient broke *his* collar bone. "If Dr. Budloo, who set it for the King the second time, had lived in this day, he would scarcely have escaped a prosecution for malpractice."

*Enlarged Tonsils.*—Miss —— aged 14. Removed by Owen's tonsil instrument. Very slight hæmorrage.

*Saturday, March* 12—*Stump made by an amputation at radio-carpal articulation.* Dr. H. made this amputation a few weeks since, and notwithstanding the fears which most surgeons entertain that amputations made through this and other large joints will not do well, this healed kindly and has left a beautiful and useful stump. The power of pronation and supination exists to a limited extent, and will probably increase. Had the amputation been made through the bones, higher up, this power would have been more completely lost.

*A conical stump of femur made by a flap amputation.*—The existence of a conical stump, Dr. H. remarked, was not in itself evidence of a bad amputation. It might result from tardy adhesion, violent inflammation, spasmodic retraction of muscles &c.

Several artificial legs, manufactured by Mr. Bodine, of Buffalo, were shown to the class. They were well made, combining the principal excellencies of artificial limbs in an eminent degree, symmetry, freedom of motion and lightness.

*Painful Cicatrix.*—E. C. aged 52 years, nervous temperament, lost the last phalanx of the thumb of left hand three years since ; was *torn* away. When it was dressed the flaps seemed ample. The wound was closed in three or four weeks, and in the mean time it was not painful.

Five weeks after the accident, a pain suddenly attacked the left shoulder, which was very intense and shot down the arm and hand. Bleeding did not alleviate it, nor was he relieved of the pain until he had taken enormous quantities of belladonna. He thus escaped narrowly from trismus and he has ever since been afflicted with pains and spasms in the arm and hand extending into the cicatrix at the end of the stump. The cicatrix is very tender, and when touched produces a slight spasm of the arm.

He is recommended to submit to an amputation of the stump, since medication would not be likely to succeed, nor would simple incision of the cicatrix, or even destruction of it by the heated iron be as likely to succeed as amputation. A similar case was referred to where Dr. H. had amputated with complete success.

*Varus, third degree, of right foot.*—In ant child of D. Hoag, of Hamburgh, aged four months. Cut tendo achilles and applied a tin shoe.

*Wednesday, March* 16.—*Epilepsia from fracture of the skull.*— —— of Buffalo aged about 25 years, was trephined for a fracture of the skull when twelve years old, by Dr. White of Cherry Valley. Has been well until within two or three years, since which time he has had epileptic convulsions frequently.

*Epilepsia, from fracture of the skull.*—Mr. Whiteham, from Hamburg, aged 35 years. Five years since was hit by a falling tree over the organ of veneration. Fourteen pieces of bone were removed. There is now a large depression at the seat of fracture. Within two or three years he has had epileptic convulsions.

Dr. H. recommended trephining in each case as a *final* resort.

Two cases of *enlargement of the submaxillary gland*, one of which had resulted in the formation of a salivary fistula.

Both to be treated by the use of iodine and by low diet. In the case of the fistula an operation may become necessary.

*Goitre.*—German girl, aged about twenty. Iodine to be used perseveringly, both internally and externally. She is to have air, exercise and good diet, and to bathe twice a day in cold water.

*Encysted Tumor* at inner canthus of right eye; size of a walnut. Dr. H. extirpated it; was found to be closely adherent to the outer wall of the lacrymal sac.

*Gonorrhœa Virulenta.*— ——, aged 22 years, contracted the disease about eight weeks since. Not much smarting at present; discharge thick and yellow. Ordered an injection of ℞—Nitras argent. gr. i., Aqua pura ℥i, to be used twice a day: also a mixture, ℞—Balsam Copaib. ℥ ii, Spts. Nit. Dulc. ℥ iii, Tr. Opii. ʒ i, of which he is to take a teaspoonful three times per day.

*Hydrocele.*— —— aged 64 years from Hamburg. The hydrocele has existed several years; lately it has become painful. Dr. H. advised him first to have the palliative operation made, so that the condition of the testis could be better determined, and after a few weeks, when it again filled up, the radical operation might be made.

It was opened with a common bistoury and discharged about a pint of straw colored serum. Testis found to be healthy.

*Amputation of finger under the influence of chloroform.*—B. a sailor, had his left hand and fingers badly crushed by a heavy timber. He was suffering intensely, and Dr. H. administered to him ʒiii of chloroform, sufficient to produce complete insensibility. The patient now turned his head over his shoulder and appeared like a man in a fit of apoplexy; the breathing was stertorous, and the eyes turned up and fixed. The effect passed off however before the operation was quite completed. Dr. H. is not an advocate for the frequent use of anaesthetic agents in surgical operations, and is of opinion that effects like these cannot be produced without hazard to the life of the patient. Wm. W.

